# Supplementary figures and images for: Nano-hydroxyapatite improves intestinal absorption of acetazolamide (BCS Class IV drug)–but how?
Source: PLoS One. 2022 May 19;17(5):e0268067. doi: 10.1371/journal.pone.0268067 (PMC9119549; doi:10.1371/journal.pone.0268067)

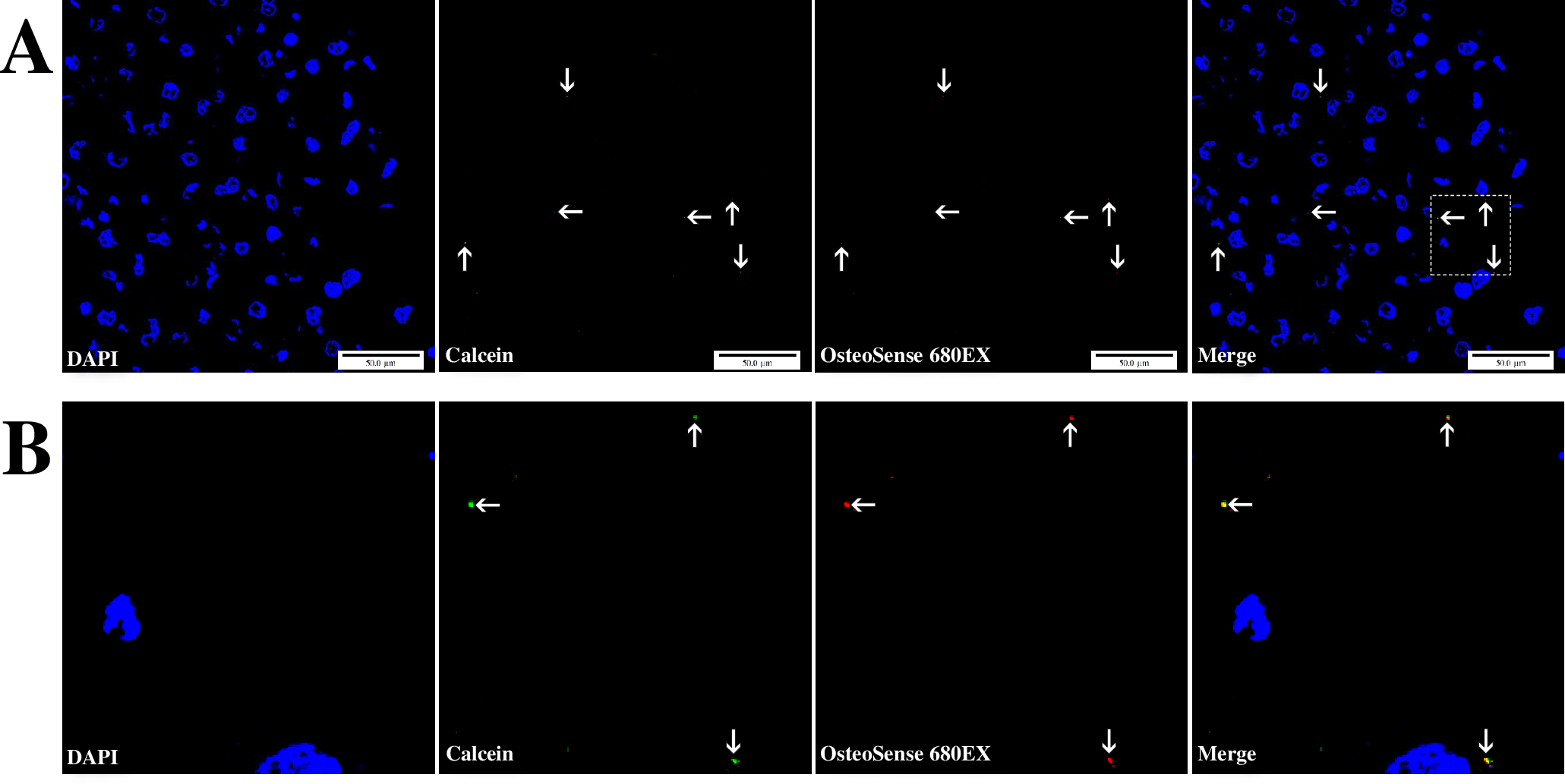

Supplement: S1 Fig — (A) Photos show the fluorescence observed at each wavelength for DAPI, Calcein, OsteoSense 680EX, and Merge, respectively. Arrows indicate the location of the nano-HAP particles seen with fluorescence. The region surrounded by dashed lines in the merge image has been enlarged in S1B Fig. (B) Enlarged images of S1A Fig. (TIF) [file pone.0268067.s001.tif]
