# Supplementary material for: Nano-hydroxyapatite improves intestinal absorption of acetazolamide (BCS Class IV drug)–but how?
Source: PLoS One. 2022 May 19;17(5):e0268067. doi: 10.1371/journal.pone.0268067 (PMC9119549; doi:10.1371/journal.pone.0268067)
Supplement: Attachment — Submitted filename: Comments.docx [file pone.0268067.s003.docx]

The authors have presented very good findings with experimental data in the manuscript. The authors are appreciated for the way of explanation given in this manuscript. This manuscript can be publishable after minor revisions.

1) Relevance of line number 55-60 with context of present study
2) In line number 72, mention name techniques. Include the following references for the solubility enhancement techniques in line number 72:
a) Diwan, Rimpy, Punna Rao Ravi, Nikita Shantaram Pathare, and Vidushi Aggarwal. "Pharmacodynamic, pharmacokinetic and physical characterization of cilnidipine loaded solid lipid nanoparticles for oral delivery optimized using the principles of design of experiments." Colloids and Surfaces B: Biointerfaces 193 (2020): 111073.
b) Diwan, Rimpy, Punna Rao Ravi, Shubham Ishwar Agarwal, and Vidushi Aggarwal. "Cilnidipine loaded poly (ε-caprolactone) nanoparticles for enhanced oral delivery: optimization using DoE, physical characterization, pharmacokinetic, and pharmacodynamic evaluation." Pharmaceutical Development and Technology 26, no. 3 (2021): 278-290.
c) Diwan, Rimpy, Shareef Khan, and Punna Rao Ravi. "Comparative study of cilnidipine loaded PLGA nanoparticles: process optimization by DoE, physico-chemical characterization and in vivo evaluation." Drug delivery and translational research 10, no. 5 (2020): 1442-1458.
3) Author can mention briefly the preparation of nano-HAP and the source of micron level HAP used to synthesize nano-HAP (line no. 103) ? Did author extract the micron level HAP from teeth or bone in the lab?
4) The author can include the brief procedure of coating of nano-HAP over the AZ particles.
5) Mention the test solution in line 115. Is it phosphate buffer pH 6.8?
6) What is the AZ weight in the 900 mg of AZ/HAP formulation taken for solubility (line 117)?
7) What is the anticipated mechanism of solubility improvement with nano-HAP ?
8) What is the reason of adjusting the AZ concentration to a final concentration of either 540 ppm or 2,240 ppm on mucosal side in Ussing chamber?
9) Did author check the solubility of physical mixture of AZ/HAP?
10) The author should show the error bars in Figure 3A – solubility of AZ alone.
11) What could be the anticipated reason for huge standard deviation in blood concentration of AZ in AZ/HAP formulation at 30 min.?
12) Line 547, author mentioned HAP is known to function as a buffer in a wide pH range. Why AZ solubility is low in DTS 2 although HAP is good buffering agent? What is pKa value of HAP?
13) The authors could have quantified the AZ concentration in the intestine after the oral administration.
14) In authors’ experiment using NPP tissue in the Ussing chamber, at a high level of initial AZ concentration, AZ permeability was significantly higher for the AZ/HAP formulation than for AZ alone, while at a lower level of initial concentration, no significant difference in AZ permeation between the two groups was observed. Why at a high level, AZ permeability is more?
